# Coping with pain among adults with chronic tic disorders

**DOI:** 10.3389/fpsyg.2025.1537088

**Published:** 2025-07-30

**Authors:** Agnieszka Małek

**Affiliations:** Chair of Clinical Psychology, Development and Education, Faculty of Social Science, Institute of Pedagogical Sciences, University of Warmia and Mazury in Olsztyn, Olsztyn, Poland

**Keywords:** Gilles de la Tourette's syndrome (GTS), Persistent (Chronic) Motor Tic Disorder (CMTD), tic, YGTSS, chronic pain, pain intensity, pain coping strategies

## Abstract

**Introduction:**

Tic spectrum disorders—Gilles de la Tourette's syndrome (GTS) and Persistent (Chronic) Motor Tic Disorder (CMTD)—are neurodevelopmental conditions characterized by recurrent motor and/or vocal tics persisting for at least 1 year. While tics are the primary symptom, pain related to tics is an important yet often overlooked aspect of these disorders. Pain can result from the repetitive nature of tics, leading to muscle strain, joint stress, or even injury due to the forceful execution of movements. Additionally, pain resulting from rapid and repeated movements or vocalizations can contribute to chronic discomfort, significantly affecting daily functioning and quality of life. Despite its impact, tic-related pain is not always addressed in clinical practice, leaving individuals struggling to find effective management strategies. The aims of this study were to assess tic-related pain, pain coping strategies and beliefs, and to investigate whether individuals experiencing pain seek professional help and how effective they perceive such interventions to be.

**Methods:**

A total of 76 participants diagnosed with GTS and CMTD participated in the study. The following scales were used: Yale Global Tic Severity Scale (YGTSS), Visual Analog Scale (VAS), Short-Form McGill Pain Questionnaire-2 (SF-MPQ-2), Pain Coping Strategies Questionnaire (CSQ), Beliefs Questionnaire for Pain Control (BPCQ), and a survey containing demographic and health data and questions about pain management and its effectiveness.

**Results:**

73.7% of participants reported tic-related pain; almost all of them declared pain located in more than one part of the body. Affective pain domain was the highest scored by both men and women. Coping self-statements was the most common coping strategy chosen by men, whereas catastrophizing was preferred by women. Respondents rated internal factors as the most important in pain management and external factors, particularly medical professionals, as the least important. This aligns with their experience, as fewer than one in five found professional interventions effective in relieving pain.

**Conclusion:**

Pain should be recognized as a common comorbid aspect of tic disorders and therefore both pharmacological and non-pharmacological interventions should include pain management. Chronic pain can significantly impair functioning in all areas of life and recommendations for the management of tic-related pain need to be developed.

## 1 Introduction

Gilles de la Tourette's syndrome (GTS) and Persistent (Chronic) Motor Tic Disorder (CMTD) are neurodevelopmental disorders characterized by the presence of one or more motor and/or vocal tics for at least 1 year (American Psychiatric Association, 2013). Typical onset of tics is between 4 and 6 years of age, with the most severe intensification between 10 and 12 years of age (Bloch and Leckman, 2009). Tics are defined as sudden, rapid, recurrent, non-rhythmic movements or vocalizations. GTS affects between 0.3 and 1% of the population, more often in children than in adults (Levine et al., 2019; Szejko et al., 2022), and the prevalence of CMTD ranges from 0.5 to 1.65% (Scharf et al., 2015). The etiology of chronic tic disorders is complex and multifactorial, involving both genetic and neurobiological mechanisms. Genetically, Tourette syndrome is understood to follow a polygenic pattern of inheritance, as supported by genome-wide association studies (GWAS) indicating a polygenic genetic architecture, with numerous common variants explaining a large proportion of heritability (Yu et al., 2019). In parallel, neurobiological models emphasize the role of dysfunction within corticostriatal circuits (Ganos et al., 2013). Because of the lack of evidence about the different origins of both GTS and CMTD, the term “tic spectrum disorders” is proposed (Müller-Vahl et al., 2019). It should be emphasized that patients with GTS exhibit a more severe clinical profile compared to those with CMTD. Individuals with GTS present with higher tic severity, a greater prevalence of complex motor tics (including copropraxia and echopraxia), as well as a higher level of psychiatric comorbidity.

It is also well-established that tic disorders frequently co-occur with various psychiatric comorbidities. Up to 90% of individuals with GTS are affected by at least one of these conditions (Robertson et al., 2017; Sambrani et al., 2016), which include attention-deficit/hyperactivity disorder (ADHD), obsessive-compulsive disorder (OCD), rage attacks, self-injurious behavior (SIB), depression, anxiety disorders, sleep disturbances, and learning difficulties (Swain et al., 2007). Tic disorders and comorbid disorders affect physical, social, occupational/academic, and psychological functioning (Cavanna et al., 2013; Evans et al., 2016; Solís-García et al., 2021). Another factor that may contribute to this impairment is chronic pain that people with tics may experience.

Pain has been reported in both children and adults with tic disorders. As many as 60% of children with different types of tic disorders may experience pain associated with involuntary movements or vocalizations (Lavenstein et al., 2016; Małek, 2022). In the adult population, 60% report tic-related pain sensations (Müller-Vahl et al., 2010). Pain interferes with the functioning of individuals in many areas of life, primarily affecting mood, sleep, and general enjoyment of life (Taylor et al., 2022). Pain caused by tics is a common reason for the decision to initiate pharmacological treatment (Cha et al., 2012).

Pain, both acute and chronic, is one of the most commonly experienced symptoms accompanying many medical conditions (Rice et al., 2016). According to the International Association for the Study of Pain (IASP), pain is “an unpleasant sensory and emotional experience associated with, or resembling that associated with, actual or potential tissue damage. (…) Pain is always a personal experience that is influenced to varying degrees by biological, psychological, and social factors” (IASP, 1994). It is called chronic pain if it lasts for more than 3 months. Chronic pain is a common condition, affecting an estimated 20% of the world's population (Treede et al., 2019). Chronic pain often leads to the perpetuation of abnormal responses in the central nervous system, resulting in changes in brain function, structure, and chemistry, which in turn can contribute to the development of secondary conditions such as depression (Borsook, 2012).

Both pharmacotherapy and psychological interventions can help reduce tic-related pain (Hollis et al., 2016). Relaxation (Tilling and Cavanna, 2020), mindfulness-based approaches (Reese, 2018), and other self-management techniques have been recognized as helpful in chronic pain relief, including tic-related pain. However, a recent study involving participants from 18 countries in Europe, the Americas, Australia, and New Zealand suggests that many people with tics do not receive adequate medical support (Taylor et al., 2022).

Previous studies on tic-related pain have not included adults from Poland, so it is reasonable to investigate the phenomenon in this population as well. To date, only one study on tic-related pain has been conducted in Poland, and it focused exclusively on children (Małek, 2022). The aims of the study were to assess pain caused by tics, pain coping strategies, and individual beliefs about pain control. We aimed to explore whether people with tic-related pain seek professional help, and, more importantly, whether this intervention helps to reduce pain.

## 2 Materials and methods

The data were collected in July 2023. The sample consisted of members of residential support groups for adults with tics, operating in several locations in Poland. These were individuals who were attending ongoing support group meetings at the time of the study. Consequently, the sample size was determined by the number of individuals available and willing to participate, rather than through formal sample size calculation or power analysis. All participants were familiarized with the purpose of the study and agreed to participate in the project. The inclusion criteria were as follows: diagnosis of GTS or CMTD made by a neurologist according to the DSM-5 criteria, age 18 and over. Participants were asked to complete a set of questionnaires provided in paper form, with no time limit for completion. In addition, one of the research tools (YGTSS) included a one-on-one interview with the participant, conducted online within a week after the paper questionnaires were completed. Participants could cancel their participation at any time without providing a reason.

The Polish adaptation of the Yale Global Tic Severity Scale (YGTSS) (Leckman et al., 1989; Stefanoff et al., 2005) was used to assess tics by their: type, number, frequency, severity, and complexity of tics, as well as the general impairment of everyday functioning. Responses are scored on a scale from 0 to 5; with 100 points being the maximum amount possible to obtain. Higher scores obtained by the individual indicates greater severity of the tic disorder and more significant impairment of daily life.

The study used the Visual Analog Scale (VAS) to score present and worst tic-related pain, and localization of pain caused by tics. Pain intensity dimensions are scored from 0 (not hurting/no discomfort/no pain) to 10 (hurting a whole lot/very uncomfortable/severe pain).

Pain descriptor was also asked based on Polish version of The Short-Form McGill Pain Questionnaire-2 (SF-MPQ-2) (Dworkin et al., 2009). This questionnaire is used to measure the quality as well as the intensity of pain and allows to evaluate both neuropathic and non-neuropathic pain. Three of four subscales consist of sensory descriptors, and the fourth consists of affective (i.e., emotional) descriptors.

To assess coping with pain, Polish version of Pain Coping Strategies Questionnaire (CSQ) (Rosenstiel and Keefe, 1983) was used. CSQ allows the following strategies in pain management to be assessed: diverting attention, reinterpreting pain sensations, catastrophizing, ignoring pain sensations, coping self-statements, praying or hoping, and increasing activity level. The higher the score on each scale, the higher the tendency to use that particular pain management strategy; multiple strategies can be used simultaneously.

For pain control assessment, the Polish adaptation of the Beliefs Questionnaire for Pain Control (BPCQ) (Skevington, 1990) was used. It allows to measure the strength of individual beliefs about controlling pain personally (internal factors), through the influence of physicians (the strength of others), and through random events.

A survey was administered to gather demographic and health data, including the type and number of comorbidities. The second part of the survey was directed at respondents who reported that tics cause them pain. It inquired about the physicians or other medical professionals consulted for tic-related pain relief, the interventions they recommended, and the participants' assessments of effectiveness and sustainability.

Statistical analysis: all analyses were performed using IBM SPSS Statistics software version 27.0 (IBM SPSS Statistics for Windows, Armonk, NY, USA). Non-parametric tests were used because the variables were not normally distributed.

## 3 Results

Seventy-six participants took part in the study−38 men and 38 women aged 18–51years (M = 31.34, SD = 10.17). Thirty-one men and 26 women were diagnosed with GTS, while 7 men and 12 women were diagnosed with CMTD. Participants diagnosed with Persistent (Chronic) Vocal Tics did not enroll. The time since diagnosis ranged from 2 to 48 years, the average duration of the tic disorder in the study group being 20 years and 8 months (SD = 12.98). Demographic characteristics of the sample is shown in Table 1.

**Table 1:** Demographic characteristics of the sample (*n* = 76).

		***N* (%)**
Gender	Male	38 (50%)
	Female	38 (50%)
	Other/rather no to say	0 (0%)
Diagnosis	GTS	57 (75%)
	CMTD	19 (25%)
	CVTD	0 (0%)
	ADHD	10 (7.6%)
Comorbidities	ASD	6 (7.9%)
	OCD	16 (21%)
	Depression	22 (28.9%)
	Anxiety	26 (34.2%)
	Eating disorders	6 (7.9%)
	Sleep disorders	20 (15.2%)
	SLD	7 (9.2%)
	Other	8 (10.5%)
	None	8 (10.5%)
Declaring tic-related pain	Male	26 (34.2%)
	Female	30 (%)

ADHD, Attention-Deficit/Hyperactivity Disorder; ASD, autism spectrum disorder; OCD, Obsessive-Compulsive Disorder; SLD, specific learning disorder.

Participants were also asked to assess tics severity according to YGTSS. Total scores (the maximum being 100 points) ranged from 5 to 78, with an average score of 38.67 ± 16.00. Male participants had a total score range of 5–78 and an average of 40.95 ± 19.31, while female participants had a score range of 8–71 and an average of 36.39 ± 18.67. The YGTSS total score in the group of participants reporting tic-related pain ranged from 7 to 78, with an average score of 42.63 ± 16.77. The average score of the men in this group was 45.81 ± 17.20, while for women it was 39.87 ± 16.17 (Table 2).

**Table 2:** Yale Global Tic Severity Scale (YGTSS) (range 0–100).

	**Participants**	**Men, median (mean, *SD*)**	**Women, median (mean, *SD*)**
YGTSS	Total	Total (*n* = 38): 38.50 (40.95, 19.31)	Total (*n* = 38): 38.00 (36.39, 18.67)
		GTS (*n* = 31): 38.00 (40.26, 20.84)	GTS (*n* = 26): 38.50 (34.54, 19.02)
		CTD (*n* = 7): 42.00 (44.00, 10.71)	CTD (*n* = 12) 37.00 (40.42, 18.01)
	Declaring pain	Total (*n* = 26): 46.50 (45.81, 17.20)	Total (*n* = 30): 39.50 (39.87, 16.17)
		GTS (*n* = 19): 47.00 (46.47, 19.26)	GTS (*n* = 20): 43.00 (39.85, 17.17)
		CTD (*n* = 7): 42.00 (44.00, 10.71)	CTD (*n* = 10) 37.00 (39.90, 14.85)

## 4 Pain severity

Tic-related pain (past or present) was reported by 56 of 76 individuals with GTS/CMTD (73.7%) taking part in this study. Twenty-six men (19 diagnosed with GTS, 7 of them with CMTD) and 30 women (20 diagnosed with GTS, 10 of them with CMTD) demonstrated pain. A scale from 0 to 10 was used to assess pain intensity, where 0 means no pain, 1–3 indicates mild pain, 4–6 moderate pain, 7–10 severe pain. To assess whether there is a difference between men and women in pain intensity assessment, both present and past, the Mann-Whitney U test was used. There was no statistically significant difference between groups (Table 3).

**Table 3:** Present and worst pain on VAS (range 0–10).

**Pain scores**	**Men (*n* = 26), median (mean, *SD*)**	**Women (*n* = 30), median (mean, *SD*)**	***p***
Present pain	Total: 3.0 (3.3, 1.3)	Total: 2.5 (2.6, 1.8)	Total: 0.092
	GTS: 3.0 (3.2, 1.4)	GTS: 2.5 (2.8, 1.8)	GTS: 0.411
	CTD: 3.0 (3.4, 1.0)	CTD: 2.5 (2.4, 1.7)	CTD: 0.315
Worst pain	Total: 4.0 (4.3, 1.5)	Total: 4.0 (4.3, 2.1)	Total: 0.743
	GTS: 4.0 (4.6, 1.5)	GTS: 5.0 (4.6, 1.9)	GTS: 1.000
	CTD: 4.0 (3.6, 1.0)	CTD: 3.5 (4.2, 2.5)	CTD: 0.669

*p* < 0.05.

In both present and worst pain ratings, participants with self-reported comorbid depression on the survey had lower mean scores (2.4 ± 1.2 and 4.1 ± 1.2, respectively) than those who did not report depression as a comorbid condition (2.8 ± 1.7 and 4.5 ± 2, respectively). When comparing the mean scores in men and women, it was found that men with self-reported depression rated the worst pain higher than those without: GTS/CMTD + depression: 2.3 ± 1.0 and 4.5 ± 0.6, respectively; GTS/CMTD without depression: 3.2 ± 1.0 and 4.3 ± 1.0, respectively. In contrast, among women, the mean score for current pain was slightly higher among participants who self-reported depression than among those who did not (GTS/CMTD + depression 2.4 ± 1.4 and 3.9 ± 1.5, respectively; GTS/CMTD without depression: 2.3 ± 1.0 and 4.5 ± 0.6). There were no significant statistical differences between the groups.

Significant correlations (Spearman's rho) were found between present pain and tic severity, and worst pain and tic severity only in female respondents, especially those with GTS. The correlations between the scores of pain intensity and tic severity are reported in Table 4.

**Table 4:** The correlations between the scorings of pain intensity and tic severity.

**Gender**	**Present pain**	**YGTSS**	**Worst pain**	**YGTSS**
Men	Total (*n* = 26)	0.189	Total (*n* = 26)	0.179
	GTS (*n* = 19)	0.206	GTS (*n* = 19)	0.159
	CTD (*n* = 7)	−0.095	CTD (*n* = 7)	0.286
Women	Total (*n* = 30)	0.540^**^	Total (*n* = 30)	0.363^*^
	GTS (*n* = 20)	0.577^**^	GTS (*n* = 20)	0.153
	CTD (*n* = 10)	0.057	CTD (*n* = 10)	0.472

* *p* < *0.05*,

** *p* < *0.01*.

## 5 Location of pain

89.29% of respondents reported pain in more than one part of the body, and the locations of the pain were consistent with the tics reported in the YGTSS. The pain mostly involved the following parts of the body: neck (53.57%), eyes (42.86%), back (39.29%), shoulders (28.57%), abdomen (25%), lips (17.86%), and joints (especially the temporomandibular joint −17.86%). 7.14% of respondents each indicated that pain caused by tics was related to the forehead and forearms, while pain of nose, palms, and thighs were reported by 3.57% each.

## 6 Pain descriptors

Pain descriptors were also asked to assess both pain domains and the severity of different types of pain sensations. Both men and women with tic disorders obtained the highest mean scores on the affective pain domain (5.00 ± 2.14 and 5.23 ± 2.42, respectively). Male participants scored lower on intermittent (2.86 ± 1.21) and continuous pain (2.79 ± 1.37); the lowest mean scores were obtained on the neuropathic domain (2.17 ± 0.75) (Table 5). In contrast, women's scores on this domain were the second highest of all (3.45 ± 2.25). Female participants scored the lowest on the intermittent domain (2.78 ± 1.56) and slightly higher on the continuous domain (2.94 ± 1.56). No statistically significant differences were found between men and women in the assessment of specific domains of pain.

**Table 5:** Pain descriptors (in percentages) and pain domains score (in points) by men and women.

**Pain domain**	**Pain descriptors**	**Men**		**Women (%)**		***p***
		**%**	**Score: total (M**, ***SD*****)**	**%**	**Score: total (M**, ***SD*****)**	
Continuous	Throbbing	14.28	22	7.14	14	
	Cramping	10.71	18	17.86	26	
	Gnawing	0	0	3.57	2	
	Aching	14.28	30	21.43	46	
	Heavy	10.71	8	14.28	12	
	Tender	0	0	0	0	
			Total: 78 (2.79, 1.37)		Total: 98 (2.94, 1.56)	0.799
Intermittent	Shooting	7.14	16	0	0	
	Stabbing	10.71	14	17.86	26	
	Sharp	3.57	4	3.57	2	
	Splitting	3.57	6	0	0	
	Electric-shock	0	0	3.57	10	
	Piercing	0	0	7.14	12	
			Total: 40 (2.86, 1.21)		Total: 50 (2.78, 1.56)	1.000
Neuropathic	Hot-burning	17.86	20	14.28	14	
	Cold-freezing	0	0	0	0	
	Pain caused by light touch	0	0	0	0	
	Itching	0	0	0	0	
	Tingling or “pins and needles”	3.57	6	10.71	18	
	Numbness	0	0	14.28	44	
			Total: 26 (2.17, 0.75)		Total: 76 (3.45, 2.25)	0.350
Affective	Tiring-exhausting	21.43	70	28.57	108	
	Sickening	0	0	0	0	
	Fearful	7.14	8	10.71	14	
	Punishing-cruel	0	0	7.14	14	
			Total: 78 (5.00, 2.14)		Total: 136 (5.23, 2.42)	0.916

*p* < 0.05.

## 7 Pain coping strategies

Analysis of the data revealed that women were significantly more likely to use a strategy of distraction, catastrophizing, praying or hoping, and increasing activity level. The most common pain coping strategies used by men were coping self-statements and increasing activity level. Both men and women were least likely to use the strategy of switching from painful to non-painful sensations, e.g., “I just think of pain as some other sensation, such as numbness.” Pain coping strategies used by both male and female participants are reported in Table 6.

**Table 6:** Pain coping strategies.

**Pain coping strategy**	**Men (*n* = 26), median (mean, *SD*)**	**Women (*n* = 30), median (mean, *SD*)**	***U***	***p***
Diverting attention	6.00 (9.54, 8.14)	14.00 (15.87, 7.70)	310	0.001^*^
Reinterpreting pain sensations	6.00 (7.69, 5.30)	9.00 (10.93, 7.98)	271.5	0.080
Catastrophizing	7.00 (8.54, 7.62)	25.00 (20.13, 11.31)	296	0.001^*^
Ignoring pain sensations	10.00 (9.85, 7.63)	10.00 (11.53, 9.79)	214	0.843
Coping self-statements	13.00 (14.38, 4.79)	16.00 (16.27, 9.84)	221	0.391
Praying or hoping	6.00 (9.00, 7.87)	19.00 (16.27, 11.16)	255	0.021^*^
Increasing activity level	11.00 (12.38, 7.32)	17.00 (18.47, 8.42)	275	0.006^*^

* *p* < 0.05. Bold values indicate statistical significance.

No significant association was found between pain coping strategies and YGTSS (Spearman's rho). However, there was a significant association between the number of years since diagnosis and reinterpreting strategy (*p* = 0.449)—the more years since diagnosis, the more patients tried to distance themselves from the pain by not thinking of it as pain, but rather as a monotonous or warm sensation or other sensations such as numbness, by imagining that the pain is outside their body, or by pretending that the pain is not part of them.

## 8 Pain control and ability to reduce pain

The Pain Coping Strategies Questionnaire also assesses the degree of pain control, as well as the ability to reduce pain intensity in the subjective assessment of the subject. This part of the questionnaire requires separate interpretation. Respondents gave their ratings on a scale from 0 to 6 points. According to the instructions in the questionnaire, when rating the ability to control pain, 0 meant that the respondent had no control over the pain, 3 meant partial control, while a score of 6 meant that the person had complete subjective control over the pain. Analysis of the pain control scores showed that a significantly greater percentage of respondents had partial control of their pain (92.86%), while complete control of pain and complete lack of control of pain were indicated by 3.57% each (women only).

Also, in the assessment of the ability to reduce pain, a scale from 0 to 6 was used, where a score of 0 meant that the respondent could not reduce pain at all, 3 indicated partial ability to reduce pain, while 6 meant that they could reduce pain completely. Most respondents were only partially able to reduce pain (89.29%), while 10.71% were not able to reduce pain at all; none of the participants reported being able to reduce pain completely.

Statistical analysis (Spearman's rho) showed a significant association between pain control and catastrophizing and ignoring pain sensations (*p* = −0.395, *p* = 0.377, respectively)—the greater the sense of pain control, the less frequently the catastrophizing strategy is used, and greater pain control influences coping with pain by ignoring it. There was also a significant association between subjective ability to reduce pain (*p* = 0.441) and catastrophizing, with the implication that those with greater pain control were less likely to manage pain by catastrophizing.

The highest mean score was obtained for internal control, i.e., one's own ability to manage pain, among both male and female participants in the study. Participants attributed the least importance to medical interventions in reducing their pain, with slightly more importance attributed to random events and interactions independent of the patient or clinician (Table 7).

**Table 7:** BPCQ test results for men and women with GTS/CMTD.

**BPCQ subscale**	**Men (*n* = 26), median (mean, *SD*)**	**Women (*n* = 30), median (mean, *SD*)**	***p***
Internal factors	32.00 (33.69, 11.64)	25.00 (27.93, 15.83)	0.156
Power of doctors	10.00 (9.69, 2.25)	11.00 (11.53, 3.74)	0.254
Chance events	10.00 (11.46, 2.82)	13.00 (13.00, 5.71)	0.496

*p* < 0.05.

Statistical analysis also showed that there was a slight positive correlation (*p* = 0.384) between tic severity (along with overall level of impairment in daily functioning) as measured by the YGTSS and belief in the importance of chance events in the ability to control pain. In addition, a moderate correlation was found between the amount of time that had elapsed since diagnosis and the sense of control over the pain (internal factors subscale) (*p* = 0.787). However, no associations were found between the BPCQ subscales and perceived pain intensity (either current or worst pain).

## 9 Help-seeking

Eighty-six percent of participants suffering from tic-related pain reported seeking help to relieve the pain (19 men and 31 women). For help, they most often reported to their primary care physician (37.5%), neurologist (57.1%) or psychiatrist (30.3%), under whose care they were due to tic disorder or co-occurring disorders. Less frequently, they asked a physiotherapist (21.4%) or psychotherapist (12.5%) for pain-focused intervention, and two people (3.6%) used acupuncture. No one sought help from a specialized chronic pain clinic. Only 18% of those seeking professional help (6 women, 4 men) felt that the treatment they received relieved pain caused by tics, but unfortunately, the effects were only temporary or insufficient.

## 10 Discussion

This study assessed the severity of pain experienced by adults with GTS and CMTD, as well as coping strategies and pain relief interventions. Measurements of pain intensity always reflect the subjective feelings of the individual and do not always directly indicate the degree of tissue damage. Pain intensity is influenced by many factors, including duration of pain, gender, interference with activities of daily living, and level of social support (Meints and Edwards, 2018). Equally important are individual coping strategies, where coping is broadly defined as the use of behavioral, emotional, and cognitive techniques to manage symptoms of distress (Lazarus and Folkman, 1984).

Participants most commonly rated their pain as mild and moderate, with no one rating the maximum on the scale. Women scored slightly lower on average for current pain, but the scores were almost identical for worst pain. It is well-documented that both women and girls report pain more frequently and experience more severe pain than men. However, it should be noted that women in the study group had lower mean scores on the YGTSS—the lower frequency, severity, and complexity of tics may be reflected in their less disabling nature and therefore in the pain caused by tics. Furthermore, unidimensional scales are useful for monitoring acute pain, for example, postoperative pain, while for chronic pain, the perception of which is conditioned by psychosocial and emotional factors, assessment based on measurement of pain intensity is incomplete (Arendt-Nielsen et al., 2012). No significant difference was found in another study that investigated differences in tic-related pain ratings between boys and girls (Małek, 2022).

Our results did not confirm higher pain scores in participants with depression, a common occurrence that leads to poor physical and psychological function, as well as longer pain duration and more severe pain perception (Ishak et al., 2018). At the same time, it should be emphasized that information on comorbid disorders, including depression, was self-reported and not derived from the screening tools used in the study.

Participants were also asked to identify the location of pain caused by tics. The pain was most commonly cervical, followed by ocular, back, shoulder, abdominal, and joint pain. Other studies have also identified these areas of the body as affected by tic-related pain, both in adults and children (Lavenstein et al., 2016; Małek, 2022; Cha et al., 2012). Most individuals declared that pain was located in more than one part of the body.

This study also qualitatively assessed the pain experienced as a result of tics. Both men and women with tic disorders obtained the highest mean score on the affective pain domain. The observed differences in the intensity of the indications in the different domains suggest that it is this component that has a significant impact on pain perception. Female participants also scored highly on items indicating the neuropathic origin of the pain they experienced. The pathophysiological mechanisms of neuropathic pain are complex and not fully elucidated (Baron et al., 2010). In people with tics, it may be caused by damage to the structures of the nervous system, as pointed out by Riley and Lang (1989).

Coping strategies are related to the level of declared pain, adaptation to chronic pain, as well as the level of psychophysical functioning (Meints and Edwards, 2018). The study found that female participants were most likely to use a catastrophizing strategy and significantly more likely to do so than men. Our findings are consistent with the results of the meta-analysis conducted by El-Shormilisy et al. (2015), which indicated that women with chronic pain are more likely to use strategies considered maladaptive (including catastrophizing). This strategy is the least adaptive, with numerous studies suggesting more intense pain, longer sick leave, and recovery time, among others (Meints and Edwards, 2018; Meyer et al., 2009). However, according to the Communal Coping Model (Burns et al., 2015), catastrophizing pain can serve as a way of communicating suffering, soliciting social support, or gaining validation from those around them (e.g., health care professionals, caregivers), and women's use of this strategy is reinforced through social learning (Sullivan et al., 2000). In contrast, there is no consensus on the role of praying as a pain management strategy; it is indicated that this strategy can be both helpful and maladaptive (Meints and Edwards, 2018; Peres and Lucchetti, 2010). However, women also chose this strategy significantly more often than men. The self-evaluation strategy, which has been negatively associated with pain measures in other trials (Flor and Turk, 1988), was most commonly used by men, who were slightly less likely to use another of the adaptation strategies—increased physical activity. Despite studies suggesting that women are more likely to use an ignoring strategy (Keogh and Denford, 2009), no such pattern was observed in this study, although the more frequent use of a distraction strategy by women was confirmed. Experimental studies indicate that directing attention to distractors significantly reduces the intensity of pain sensations (Rischer et al., 2020). Reinterpreting pain sensations, which is considered to be associated with greater control over the pain (Haythornthwaite et al., 1998), was the least frequently chosen strategy by both men and women.

In the study presented, respondents attributed similarly low importance to the influence of doctors and medical care and chance events in pain control, and the highest importance to their own ability to control tic-related pain. These findings are consistent with patients' experiences of seeking professional help for pain management. Indeed, respondents indicated that both pharmacotherapy and psychotherapy, as well as the help of physiotherapists, had unsatisfactory results in reducing the pain experienced. These findings complement the data provided by Taylor, Anderson, and Davies (Taylor et al., 2022).

Many studies have shown that, on the one hand, it is difficult to access specialists who demonstrate an understanding of the nature of tics, and on the other hand, doctors often have both poor knowledge of tic disorders and often lack empathy (Taylor et al., 2022). Pain has not only genetic, molecular, cellular, and physiological dimensions, but also social, cultural, and psychological dimensions, making it a multidimensional phenomenon (Lombana and Vidal, 2012). Thus, all of the factors identified may contribute to tic-related pain sufferers' preference for self-management of pain, showing little trust in specialized interventions. As indicated by Paller et al. (2009), not only the above-mentioned factors, but also the way pain is reported (which is also related to social and cultural conditioning) can lead to different ratings and treatment depending on the gender of the person reporting the pain. It seems reasonable, therefore, to consider whether, regarding tic-related pain, gender may determine the treatment of pain in another context as well—the sudden increase in the number of young women with rapid onset tic behavior since the start of the pandemic is remarkable (Pringsheim et al., 2021), and the popularity of recordings of them on social media is enormous. It may have fostered the belief that “tics” could facilitate peer acceptance or even enhance popularity. This, in turn, could intersect with broader societal narratives about women experiencing tics and consequently being inadequately acknowledged by healthcare professionals.

Our study has some limitations. One is the lack of representation of individuals with Persistent (Chronic) Vocal Tic Disorder. Due to the total number of participants, caution should be exercised in generalizing the conclusions to the entire population of individuals with different types of tics. Additionally, no a priori power analysis was conducted. The sample size was not determined based on statistical criteria but was instead dependent on the availability and willingness of support group members to participate. This may limit the generalizability of the findings and the statistical power to detect small effects.

In retrospect, given the possible bias in pain rating, further studies on pain should consider rating the most common pain rather than the worst pain. Moreover, the use of multidimensional (rather than unidimensional) scales to measure tic-related pain would allow assessment of both pain severity and the impact of chronic pain on different aspects of the patient's functioning, physical activity, wellbeing, and health-related quality of life.

Furthermore, the introduction of questions about the patient's own ways of coping with pain could help to analyze in detail the effectiveness of non-pharmacological methods of tic-related pain relief.

In addition to the limitations discussed above, it is important to note that the present study focused exclusively on adults. Therefore, the findings cannot be generalized to children and adolescents with tic disorders. Given the differences in developmental stages, symptom presentation, and coping strategies, future research should specifically address tic-related pain in pediatric populations.

## 11 Conclusions

Our study revealed that pain is a common comorbid aspect of tic spectrum disorders. Sufferers of tic-related pain, which usually involves more than one part of the body, rarely receive adequate help from specialists. Therefore, they most often rely on their own abilities to cope with the pain. There is a tendency for women to use maladaptive strategies to cope with their pain, whereas men usually use strategies considered useful for pain reduction. Based on pain descriptors chosen by participants, it is reasonable to argue that pain is both nociceptive and neuropathic, which implies the need to differentiate the interventions used, both pharmacological and non-pharmacological.

These findings highlight the need for a holistic approach to pain caused by tics, as doctors, psychotherapists, and other professionals working with people with tics do not have adequate procedures to respond appropriately to the pain experienced by the patient. As noted above, numerous studies indicate that both pain and tic disorders contribute to a decline in quality of life, so patients with both conditions should receive the most comprehensive support possible to protect them from deterioration and associated impairment in physical, educational, occupational, personal, and social functioning.

## Data Availability

The raw data supporting the conclusions of this article will be made available by the authors, without undue reservation.
