# Supplementary material for: Coping with pain among adults with chronic tic disorders
Source: Front Psychol. 2025 Jul 30;16:1537088. doi: 10.3389/fpsyg.2025.1537088 (PMC12344498; doi:10.3389/fpsyg.2025.1537088)
Supplement: Supplementary file 1 [file Data_Sheet_1.pdf]

Appendix

Part II

Questionnaire on Seeking Medical Help for Pain Caused by Tics

Dear Sir/Madam,

If you indicated on the pain questionnaire that tics cause you pain, please answer the following questions:

1. **Have you sought medical or therapeutic help for tic-related pain?**

☐ Yes ☐ No ☐ I don't remember/it's hard to say.

2. **If you sought help for pain caused by tics, then to which specialist (you can choose more than one option)**

☐ Primary care physician
☐ The doctor where I am treated for tics.
  ☐ Neurologist ☐ Psychiatrist
  ☐ Other specialist (please specify)_________________
☐ Pain Management Specialist
☐ Rheumatologist
☐ Orthopedist
☐ Chiropractor
☐ Psychotherapist
☐ Physiotherapist
☐ Osteopath
☐ Other specialist (please specify) ____________________________

3. **What kind of help did you receive? (you can choose more than one option)**

☐ Pain medications (e.g., NSAIDs-nonsteroidal anti-inflammatory drugs, paracetamol)
☐ Increasing the dosage of a tic medication
☐ Muscle relaxants (e.g., baclofen, tizanidine)
☐ Antidepressants (e.g., amitriptyline, nortriptyline) or serotonin and norepinephrine reuptake inhibitors (venlafaxine or duloxetine)
☐ Antiepileptic medication (e.g., gabapentin, pregabalin)
☐ Physiotherapy

- ☐ Medical massage
- ☐ Manual therapy
- ☐ Electrostimulation (e.g., TENS-transcutaneous nerve stimulation)
- ☐ Heat or cold therapy (e.g., sauna, cryotherapy)
- ☐ Relaxation/yoga/meditation
- ☐ Acupuncture
- ☐ Psychotherapy
- ☐ Other (please specify) ________________________________

4. Has the help received been effective in relieving tic-related pain?

☐ Yes ☐ No ☐ I don't remember/it's hard to say.

5. If the help was ineffective, what might have been the reasons? (you can choose more than one option)

- ☐ The side effects of the medication were too severe
- ☐ Therapy did not work (did not relieve pain)
- ☐ The treatment was not properly tailored to my needs
- ☐ The effect was only temporary, and the pain returned
- ☐ Limited access to a specialist (e.g., due to long waiting periods or financial constraints)
- ☐ The doctor/therapist did not address my problem properly
- ☐ I did not receive proper guidance on further treatment
- ☐ Other (please specify) ________________________________

6. Would you like to share anything about your experiences with managing tic-related pain?

____

____

Thank you for completing the survey!
